# Physical Activity Trend eXtraction: A Framework for Extracting Moderate-Vigorous Physical Activity Trends From Wearable Fitness Tracker Data

**DOI:** 10.2196/11075

**Published:** 2019-03-12

**Authors:** Louis Faust, Cheng Wang, David Hachen, Omar Lizardo, Nitesh V Chawla

**Affiliations:** 1 Department of Computer Science and Engineering University of Notre Dame Notre Dame, IN United States; 2 Interdisciplinary Center for Network Science and Applications University of Notre Dame Notre Dame, IN United States; 3 Department of Sociology University of Notre Dame Notre Dame, IN United States; 4 Department of Sociology University of California Los Angeles, CA United States

**Keywords:** mHealth, fitness trackers, activity trackers, exercise, health behavior, physical activity, health, mental health, perception, social network

## Abstract

**Background:**

Moderate-vigorous physical activity (MVPA) offers extensive health benefits but is neglected by many. As a result, a wide body of research investigating physical activity behavior change has been conducted. As many of these studies transition from paper-based methods of MVPA data collection to fitness trackers, a series of challenges arise in extracting insights from these new data.

**Objective:**

The objective of this research was to develop a framework for preprocessing and extracting MVPA trends from wearable fitness tracker data to support MVPA behavior change studies.

**Methods:**

Using heart rate data collected from fitness trackers, we propose Physical Activity Trend eXtraction (PATX), a framework that imputes missing data, recalculates personalized target heart zones, and extracts MVPA trends. We tested our framework on a dataset of 123 college study participants observed across 2 academic years (18 months) using Fitbit Charge HRs. To demonstrate the value of our frameworks’ output in supporting MVPA behavior change studies, we applied it to 2 case studies.

**Results:**

Among the 123 participants analyzed, PATX labeled 41 participants as experiencing a significant increase in MVPA and 44 participants who experienced a significant decrease in MVPA, with significance defined as *P*<.05. Our first case study was consistent with previous works investigating the associations between MVPA and mental health. Whereas the second, exploring how individuals perceive their own levels of MVPA relative to their friends, led to a novel observation that individuals were less likely to notice changes in their own MVPA when close ties in their social network mimicked their changes.

**Conclusions:**

By providing meaningful and flexible outputs, PATX alleviates data concerns common with fitness trackers to support MVPA behavior change studies as they shift to more objective assessments of MVPA.

## Introduction

### Motivation

Recently labeled as 1 of 5 low-risk healthy lifestyle factors associated with reducing all-cause mortality and extending life expectancy, moderate-vigorous physical activity (MVPA) is fundamental to maintaining proper health [1]. MVPA, such as brisk walking, running, or aerobic exercise, boosts the immune system, lowers stress levels, and has been linked to the prevention of many chronic diseases including cardiovascular disease, diabetes, cancer, hypertension, obesity, depression, and anxiety [2-6]. Although a critical component of health, many individuals still lead predominantly sedentary lifestyles, increasing their risk of developing health complications [7,8].

In response, a wealth of research investigating MVPA behavior change has been conducted across all ages, ranging from longitudinal and exploratory studies to targeted interventions [9-11]. However, many of these studies have relied on subjective and intermittent measures of MVPA with surveys acting as the primary method of data collection, often administered only twice: at baseline and follow-up assessments. To leverage more objective measures of MVPA, many recent study designs have begun to incorporate wearable fitness trackers [12].

Although the mobility and minimally invasive nature of these wearable devices posit them as ideal tools for objective MVPA assessments, data generated from these devices cannot be as readily analyzed in comparison to survey data [13]. Preprocessing steps must be taken to address biases in device measurement and the highly granular nature of time series data [14].

To leverage the strengths of fitness tracker data for MVPA behavior change studies, a framework is necessary to alleviate data concerns and extract meaningful trends, which better conform to more traditional statistical analyses.

### Background

To ensure more objective measurements of MVPA, recent behavior change studies have begun to adopt wearable fitness trackers. Despite the long-term capabilities of these devices, many study designs track participants for only a limited time (typically 1 week) during assessment periods [15-20]. Through this design, the amount of MVPA performed in each assessment window can be summed and treated as any other repeated measures variable, more akin to survey responses.

Investigations into gathering new insights from these intermittent measurements have also been conducted. Sprint et al proposed a framework for automatically detecting behavior changes given 2 windows or samples of fitness tracker data [21]. The work expanded upon activity change detection to determine whether the change was significant, providing visualizations and summary data to better understand what type of change had occurred.

Many novel approaches exist for detecting behavior change; however, their focus is often outside of MVPA, such as investigating smart home sensors for eldercare monitoring and security threats [22,23]. Although other works focus on general change-point detection in time series, they lack the insights necessary for understanding physical activity (PA) data [24]. As such, limited work exists that focuses specifically on using highly-granular long-term data to monitor MVPA behaviors.

### Objectives

The objective of this research was to develop a framework for assessing MVPA behavior changes using continuous and objective data provided by fitness trackers. To do so, we propose Physical Activity Trend eXtraction (PATX): a framework for preprocessing and extracting MVPA trends from heart rate (HR) data. To demonstrate how these extracted trends can support MVPA behavior change research, we provide 2 case studies, drawing comparisons to previous research and demonstrating novel behavior change analysis.

## Methods

### NetHealth Study

#### Study Design and Data

The data used in this paper come from the NetHealth study conducted at the University of Notre Dame. All procedures were fully approved by the institutional review board before distribution.

**Figure 1:**
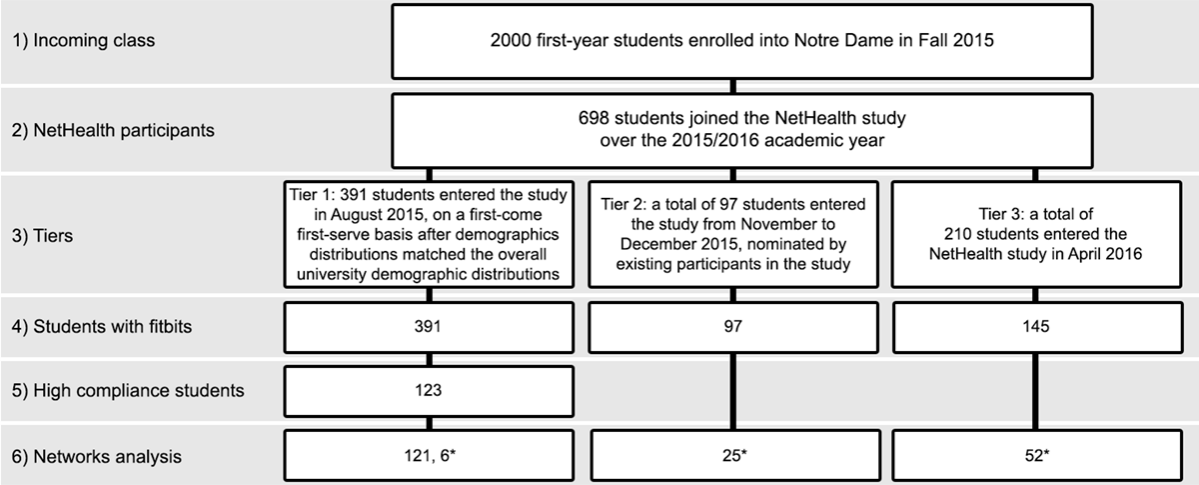
Consort diagram of NetHealth recruitment and participants selected for this analysis in this paper. Asterisks indicate participants added as additional alters for the ego-network analysis in case study 2.

Participants were recruited across 3 tiers based on when they entered the study, the process and sample numbers are outlined in Figure 1. A total of 391 Tier-1 participants were recruited via an interest survey in June 2015 and solicitations made through email and a Facebook page. Recruitment was on a first-come, first-serve basis after matching the overall demographic distributions of the university. A total of 97 Tier-2 participants were then recruited in November and December 2015, nominated by existing participants in the study. Finally, 210 Tier-3 participants entered the study in April 2016. Participants received a Fitbit Charge HR either before arriving on campus, after arrival, or in the Spring 2016 semester, depending on when they entered the study.

The NetHealth study features a collection of demographic, psychometric, social network, and health behavior data. Demographic and psychometric data were collected through surveys administered to participants once a semester. Health behaviors, PA, and sleep were captured through Fitbit Charge HRs. Participants were asked to wear their devices as much as possible and sync them every 4 to 7 days. Participants’ social networks were mapped through phone calls and short message service (SMS) text messages recorded via a smartphone app.

Fitbit data spanned 2 academic years: 2015 to 2016 and 2016 to 2017. Seasonal breaks were removed from consideration as compliance issues were most severe during these times and days were not representative of a participant’s time on campus [25]. This left a time span of 18 months with 9 months per academic year equating to roughly 4 and 1/2 months per semester.

#### Cohort Selection

Among the 698 NetHealth participants, 65 were removed from consideration as they were not issued Fitbits, with reasons ranging from participants declining them to dropping the study before the device could be issued (Figure 1, level 4). Furthermore, we chose to investigate only Tier-1 participants as Tiers 2 and 3 did not enter the study until late Fall 2015 and Spring 2016. Including sufficient data from Fall 2015 was critical to our analysis as the initial weeks in this semester served as our best proxy to understanding participants’ MVPA behaviors before entering college and fully adjusting to a different environment and forming new social networks.

Participants with extended periods of missing data were excluded to avoid biases from noncompliance. This was measured by the number of compliant days in each participant’s *least compliant* semester. A day was considered *compliant* if the participants wore their Fitbit for a minimum of 80% (19 hours). This threshold was determined by computing the total number of daily records that would be eligible for analysis at each minimum daily wear time. Sweeping from 100% to 0% with a step size of 10, we found 80% acted as an inflection point for minimum daily wear time, making 77% of all daily records eligible, after which any gain in the cumulative number of records rapidly diminished. Furthermore, this allowed for consistency with other NetHealth studies including subjects with at least 80% of compliance, as this threshold was noted as appropriate for accurate estimation of PA and sleep [25].

**Figure 2:**
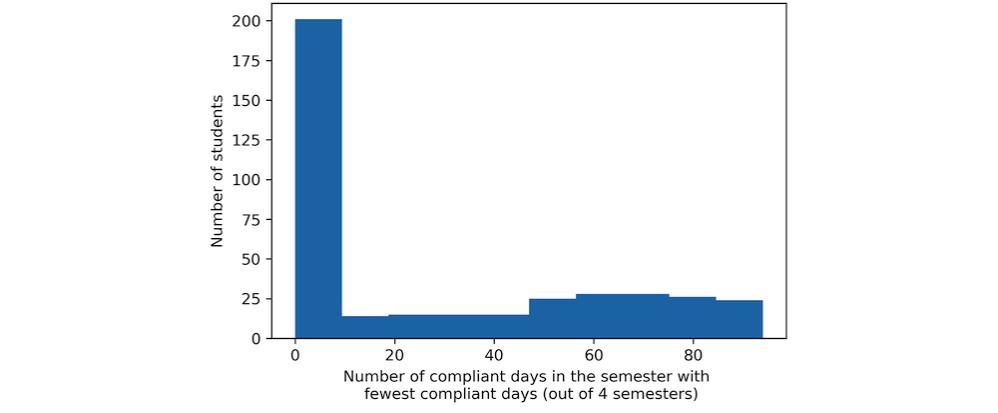
Distribution of participants’ number of compliant days in their least compliant semester across the 4 semesters measured.

A distribution of the number of compliant days in each participants’ least compliant semester is shown in Figure 2. A total of 182 participants had a semester of 0 compliant days, which can be attributed to participants dropping the study. Ignoring these participants, the median number of compliant days in a participant’s least compliant semester was 49, approximately *half* of the total days in a semester. A total of 123 participants were above this median of 49 compliant days per semester and were selected as the cohort for analysis in this paper (Figure 1, level 5). A demographic overview of these participants is provided in Table 1. Comparisons were made between the 123 participants included in the study and the 510 excluded, which we address in our *Limitations* section.

**Table 1:** Comparison of baseline characteristics between Fitbit participants included in this analysis and participants excluded based on compliance.

Variable		Included (n=123)	Excluded (n=510)
Age (years), mean (SD)		17.8 (0.4)	17.7 (0.5)
**Gender, n (%)^a^**			
	Male	65 (52.8)	265 (51.9)
	Female	58 (47.1)	245 (48.0)
**Race, n (%)^b^**			
	White	81 (65.8)	335 (65.6)
	Latino	21 (17.0)	57 (11.1)
	Asian	12 (9.7)	46 (9.0)
	Black	4 (3.2)	34 (6.6)
	Foreign	4 (3.2)	38 (7.4)
	Unknown	1 (0.8)	0 (0.0)
**High school, n (%)^a^**			
	Public school	64 (52.0)	253 (49.6)
	Private school	59 (47.9)	245 (48.0)
	Home school	0 (0.0)	2 (0.3)
	Other	0 (0.0)	10 (1.9)
**Parents’ income, n (%)^a^**			
	Less than $25,000	3 (2.4)	27 (5.2)
	US $25,000-$49,999	10 (8.1)	29 (5.6)
	US $50,000-$74, 999	14 (11.3)	38 (7.4)
	US $75,000-$99,999	10 (8.1)	47 (9.2)
	US $100,000-$149,999	25 (20.3)	97 (19.0)
	US $150,000-$199,999	12 (9.7)	55 (10.7)
	US $200,000-$249,999	10 (8.1)	50 (9.8)
	US $250,000 or more	34 (27.6)	143 (28.0)
	Unknown	5 (4.0)	24 (4.7)

^a^N=633, 100%

^b^N=632, 99.8%

**Figure 3:**
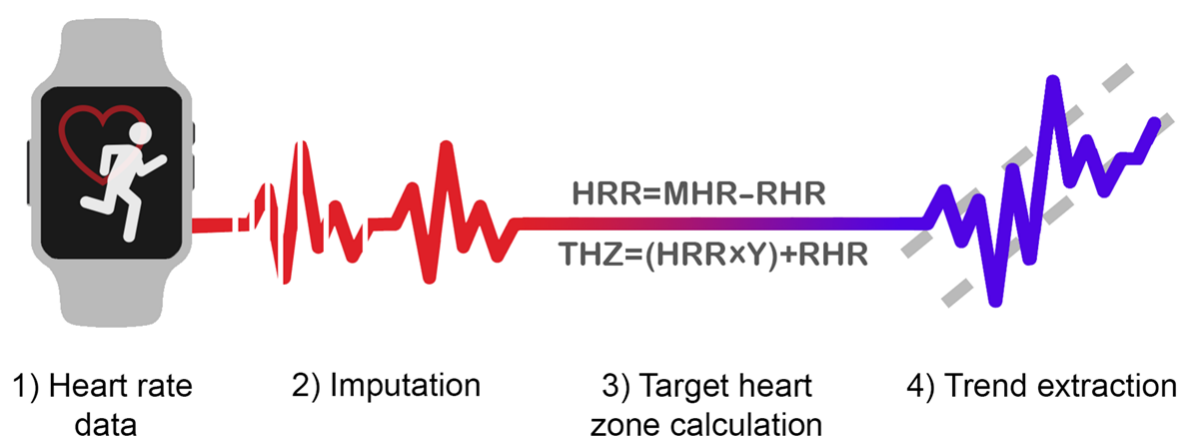
Overview of Physical Activity Trend eXtraction (PATX): a framework for moderate-vigorous physical activity trend extraction from raw heart rate data.

### Physical Activity Trend eXtraction

To accurately capture trends in participants’ MVPA, we propose PATX, a framework for preprocessing HR data to extract meaningful trends in behavior. First, we justify using HR as our input for this framework and then walk through each component of PATX: imputation, target heart zone calculation, and trend extraction (Figure 3).

#### Inputs

Minute-by-minute HR data were used for determining MVPA. HR data were favored over steps as steps are a widely involuntary measure of one’s PA. Many factors may influence steps over time, especially dynamic environments such as a college campus where destinations within daily routines are subject to change with each semester. Furthermore, steps alone cannot guarantee meeting the intensity or bout length guidelines necessary to confer health benefits, as such, benchmarks including the 10,000 steps per day goal have fallen under scrutiny [26].

Through HR data, PATX captures when individuals enter their target heart zone: a personalized HR range entered when an individual is performing MVPA [27]. To demonstrate the disparity between *minutes spent in the target heart zone* and *steps*, we conducted 2 correlation tests between these measures. The first test considered only days when users logged *steps,* and the second considered only days when users *entered their target heart zone*. For the *steps* only test, the Spearman correlation was minimal (*r*_*s*_=.1) but was far stronger in the *target heart zone* only test (*r*_*s*_=.85). These comparisons suggest that more time spent performing MVPA (being in the target heart zone), on average, results in more steps; however, more steps do not always result in more MVPA.

Despite favor over steps, HR is not a perfect measure of MVPA. Earlier studies have shown many wearable trackers, such as Fitbit Charge HRs, are affected by systematic errors and overestimation of certain HR zones [28,29]. To account for these errors, PATX includes 2 preprocessing steps: imputation and heart zone calculation.

#### Imputation

**Figure 4:**
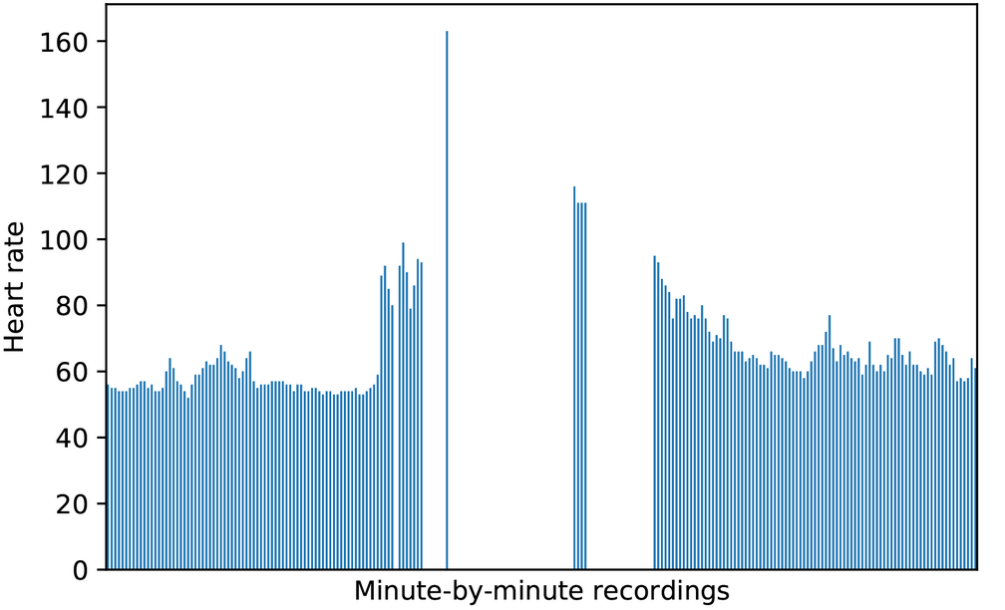
Minute-by-minute Fitbit recordings during a bout of elevated heart rate.

Apart from missing data because of noncompliance, we found a user’s HR was not always recorded during bouts of MVPA. These gaps could be attributed to moisture interfering with the readings or the band sliding outside optimal wrist placement. Such artifacts are illustrated in Figure 4: HR begins to increase, at which point data are received only intermittently, and as HR decreases, the recordings become steady, suggesting the bout of activity has ended. As Fitbit’s *cardio* and *peak* minutes do not account for these missing data, the first step in PATX was to impute such data, forming complete bouts of MVPA.

To determine the best imputation method, several algorithms were tested on complete 24-hour HR records with blocks of time set to *missing* to evaluate the accuracy of each algorithm. Incomplete 24-hour HR records were then analyzed to determine the most frequent time spans of missing data. Moreover, 1 record of daily data was pulled from each NetHealth participant at random, which had 20% of its data missing. This threshold was used as no records involved in this analysis had more than 20% of data missing as per our compliance threshold. Each record was then parsed to determine the average length of consecutive minutes that were missing. The most common missing data lengths ranged from 2 to 25 min; however, lengths as high as 150 min were also present. Given these variations, time spans of 25, 50, and 150 min were selected.

**Table 2:** Root-mean-square-error of best performing imputation method across various lengths of missing data.

Missing data length and method		RMSE^a^
**25 min**		
	Kalman ARIMA^b^	10.5
	Linear interpolation	10.9
	Kalman structural time series	10.9
**50 min**		
	Kalman ARIMA	13.2
	Linear interpolation	12.3
	Kalman structural time series	12.3
**150 min**		
	Kalman ARIMA	14.2
	Linear interpolation	13.7
	Kalman structural time series	13.8

^a^RMSE: root-mean-square error.

^b^ARIMA: autoregressive integrated moving average.

Complete 24-hour HR records were then divided into blocks of 25 min, blocks were deleted at random until roughly 20% of the record was missing. Several imputation algorithms were then executed, including linear interpolation, an autoregressive integrated moving average model with a Kalman smoother, and a structural time series model with a Kalman smoother [30]. All imputation methods were evaluated using root-mean-square error (RMSE). Tests were then repeated using block sizes of 50 and 150 min, respectively. Linear interpolation produced the smallest RMSE (Table 2) for blocks of 50 and 150 min. Given the negligible difference among the algorithms for blocks of 25 min, linear interpolation was chosen for imputing the HR data.

#### Target Heart Zone Calculation

Following imputation, target heart zones were computed for each NetHealth participant using the Karvonen formula to determine when bouts of MVPA occurred [31]. Although this formula is, at best, a proxy for determining target heart zones, we found it to be the most appropriate method given our sample’s truncated age range and limited resources for more robust measurement [32]. The formula is outlined for convenience below. Although the constant *Y* can be modified, 0.5 is typically recommended by the American Heart Association [33]:

1. Y=0.5
2. Heart rate reserve (*HRR*)=max heart rate−resting heart rate (*RHR*)
3. Target heart zone minimum=(*HRR*×*Y*)+*RHR*

Target heart zones were calculated for each user by each day to account for potential changes in resting HR over the 2 academic years.

Bouts of MVPA required participants to stay within their target heart zone for at least 10 consecutive minutes, a threshold in line with the US Department of Health and Human Services PA guidelines [34]. Although recent work shows bouts of any duration may still result in mortality benefits, at the time, there is stronger support for bouts of at least 10 min [35]. To further account for any errors in device recording or HR falling temporarily outside the target heart zone, consecutive bouts separated by 1 min outside the target heart zone were combined. Finally, bouts of MVPA were then aggregated into daily sums, which we further refer to as *target minutes*.

**Figure 5:**
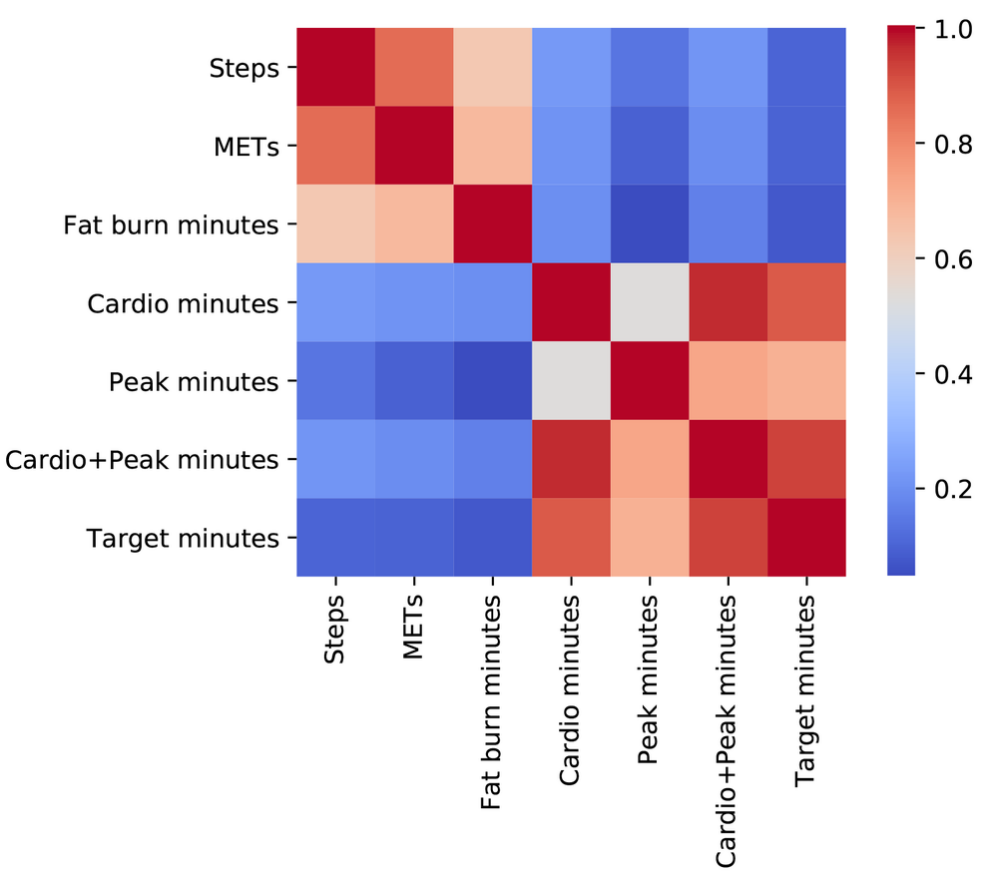
Spearman correlation matrix comparing target minutes to other physical activity measures provided by Fitbit.

In comparison to daily aggregates of PA data collected by Fitbit (Figure 5), we find target minutes correlated positively with all Fitbit measures and strongly with Fitbit's *cardio* (*r*_*s*_=.9) and *peak* (*r*_*s*_=.7) heart zone measures, which also utilize the Karvonen formula [36].

Fitbit *cardio* and *peak* minutes were then aggregated and compared with target minutes. We observed a mean absolute error of 6.20, with a session of MVPA typically resulting in 6 more target minutes than the combined *cardio* and *peak* minutes.

#### Trend Extraction

To adjust for the 18 months of data collection, participants’ daily target minutes were downsampled to sums of target minutes per month. The time series of each NetHealth participant spanned from August 2015 to April 2017, with the summer months May, June, and July withheld. The sum of target minutes for each month was then divided by the participants’ number of compliant days in that month. This was done to normalize months with breaks such as December and account for differences in participants’ compliant days per month. Trends were then extracted from each time series using additive seasonal decomposition with a frequency of 9 to account for the repeated academic years [37]. For time series of varying lengths, this frequency can be modified accordingly, or the decomposition can be replaced with a Loess smoother if no seasonality is present. A Mann-Kendall test was then performed on each extracted trend to determine whether it constituted a significant monotonic upward or downward trend [38]. Statistically significant trends were determined using a *P* of .05.

Finally, considerations were made to ensure trends were truly reflective of changes in MVPA and not of systematic differences in *when* participants were more complaint. For example, participants with a negative trend (NT) may have contributed fewer compliant days overtime, potentially capturing less MVPA. Although such a bias should be corrected for when normalizing by how many days a participant was compliant each month, to ensure a bias did not exist, Kruskal-Wallis H-tests were performed, measuring the distribution of compliant days per month across the 3 trend types, using an alpha of .05 [39,40]. No statistically significant results were found, suggesting participants from each trend group contributed a similar number of compliant days to each month.

### Case Studies

Although PATX was able to extract MVPA trends from HR data, we believe these trends alone are needless if they cannot provide meaningful insights. To evaluate this, we performed 2 case studies. The first examined the association between changes in MVPA and mental health, determining whether PATX would be consistent with earlier work. The second assessed self-perceptions of PA relative to a friend’s activity to demonstrate how PATX could support novel behavior change studies.

#### Case Study 1: Associations Between Changes in Physical Activity and Mental Health

To observe associations between trends in MVPA and mental health, participants’ depression—Center for Epidemiologic Studies Depression (CESD) Scale—and anxiety—Beck Anxiety Inventory (BAI) and State-Trait Anxiety Inventory (STAI)—screenings were evaluated along with self-reports of overall health, body-image, and self-esteem [41-43]. As a reminder, surveys containing these questions were administered once per semester.

As our focus was to assess how each participant’s response changed overtime, within-group methods were used for analysis. This included nonparametric repeated measures analysis of variance (ANOVA; Friedman test) or the Wilcoxon signed-rank test, depending on how frequently each survey question was asked. Any missing survey values were imputed using forward fill to keep the participants’ answers consistent across the missing responses.

#### Case Study 2: Assessing Self-Perceptions of Physical Activity Relative to Friend’s Activity

Our second case study focused on an exploratory analysis of how participants in these MVPA trends perceived their own levels of PA relative to their friend’s PA overtime. Survey data were again utilized with 2 questions asked: “During the current semester, how physically active would you say you are?” and “How physically active is your typical Notre Dame friend?” All comparisons were made using nonparametric repeated measures ANOVA (Friedman test).

To further explore participants’ perceptions of their friend’s PA, ego-network analyses were conducted where participants acted as the focal node (ego) of their network. The network was then populated with only that participant’s immediate friends (alters), all of whom were connected to the participant. A total of 2 participants were omitted from this analysis as they had no SMS data.

Given that only Tier-1 study participants were selected for analysis, this reduced the chances of capturing their full ego networks. To compensate, we allowed previously excluded participants to be reconsidered but *only as alters*. Since survey assessments of self and friend’s PA began in the Spring 2016 semester, we were only interested in whether the alters’ MVPA changed from this semester onward; therefore, sufficient compliance was not necessary for Fall 2015.

Compliance restrictions followed the same threshold of *at least 49 compliant days per semester*. Recall that Tier-2 participants entered the study toward the end of the Fall 2015 semester and Tier-3 participants entered near the end of the Spring 2016 semester. Due to this, Fall 2015 was ignored for Tier 2 and the Fall 2015 and Spring 2016 semesters were ignored for Tier-3 participants. Lessening these compliance restrictions added 83 more eligible participants to be considered as alters, among whom 31 were from Tiers 1 and 2, and 52 were from Tier 3.

These participants’ HR data were then processed through PATX. Tier-1 and Tier-2 participants had 13 months (Spring 2016-Spring 2017), whereas Tier-3 participants had 9 months (Fall 2016-Spring 2017). Among these participants, 9 were labeled with an NT and 2 with a positive trend (PT), these 11 participants were from Tiers 1 and 2. No Tier-3 participant was labeled with significant trends.

Finally, each ego network was limited to the top 5 strongest ties. This ensured all participants had comparably sized networks and prevented noise introduced from larger networks with weaker ties. Tie strength was measured using the total number of days 2 participants exchanged SMS text messages, with more days indicating stronger ties.

## Results

### Physical Activity Trend eXtraction

**Figure 6:**
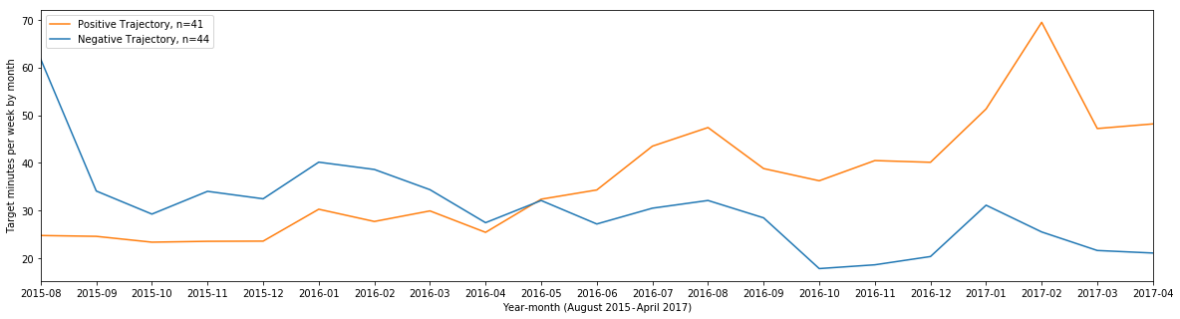
Average trend for each group from August 2015 to April 2017, which includes May, June, and July of 2016.

Among the 123 participants examined, 41 were labeled with a significant PT and 44 with a significant NT. An average time series for these trends is visualized in Figure 6. We observe that, on average, NT participants engaged in 30 min to 60 min of MVPA in a given week across their first academic year (August 2015 to April 2016) and 15 min to 30 min per week in their second academic year (August 2016 to April 2017). PT participants typically engaged in 20 min to 30 min of MVPA per week in their first academic year and 30 min to 70 min in their second.

### Case Studies

#### Case Study 1: Associations Between Changes in Physical Activity and Mental Health

Table 3 presents the findings for changes in survey responses regarding mental health across the 2 academic years. We observe that for PT participants, self-reports of health, self-esteem, and body image increased (+7%, +4%, and +6%). However, no significant results were observed for changes in anxiety or depression screenings. For NT participants, we observed a decline in self-reported health (−3%) and no significant changes in self-esteem or body image. Regarding screenings, we observed an increase in the risk of anxiety and depression (BAI +5%, STAI +8%, and CESD +9%).

#### Case Study 2: Assessing Self-Perceptions of Physical Activity Relative to Friend’s Activity

Table 4 presents the findings for changes in perception of self and friend’s PA stratified by MVPA trend. For PT participants, no significant changes were observed for the perception of their own PA; however, PT participants perceived increases in their friend’s level of PA (+4%). NT participants perceived a decrease in the level of their own and their friend’s activity respectively (−8%, −7%). These changes in perception of friend’s activity motivated our ego-network analysis.

**Figure 7:**
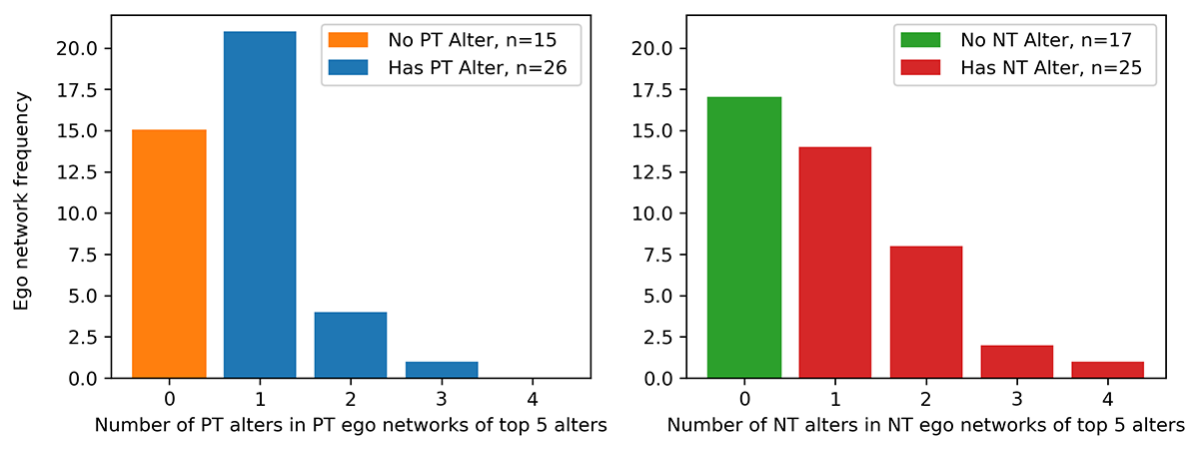
Distribution of same-trend alters in positive trend and negative trend ego networks. NT: negative trend; PT: positive trend.

For each ego network, we first examined the number of same-trend alters. Referring to Figure 7, we observed 15 PT participants with no PT alters among their top 5 strongest ties and 26 with at least one PT alter. For NT participants, we observed 17 with no NT alters and 25 with at least one NT alter. Table 5.

Partitioning each trend group into *presence of same-type alter* and *absence of same-type alter* in their ego network, we revisited the PA perception questions now stratified by these 2 conditions. Referring to Table 5, we observed that PT participants *with* PT alters did not perceive significant changes in their own or their friend’s level of PA. However, PT participants *without* PT alters perceived a significant increase in the level of their own PA (+4%) and a decrease in the level of their friend’s PA (−4%). Moving to NT participants, we observed that NT participants *with* NT alters perceived a significant decrease in their friend’s level of activity (−8%), whereas NT participants *without* NT alters perceived no significant changes in their friend's activity. Finally, we observed evidence suggesting NT students with no NT alters perceived a decrease in their own activity (-10%) and no significant changes for NT students with NT alters.

**Table 3:** Summary of descriptive statistics for moderate-vigorous physical activity trajectories based on survey responses. *Change* refers to the percent difference between the average score for the first and last survey administered.

Category		Positive MVPA^a^ Trend	Negative MVPA Trend
		Change (*P* value)	Change (*P* value)
**Self-image (higher score indicates a more positive perception)**			
	Health	+7% (.02)	−3% (<.001)
	Self-esteem	+4% (.01)	<1% (.32)
	Body image	+6% (.04)	−2% (.97)
**Mental health, risk of...(higher score indicates a higher risk)**			
	Anxiety (BAI^b^)	<1% (.60)	+5% (.01)
	Anxiety (STAI^c^)	+3.7% (.10)	+8% (<.001)
	Depression (CESD^d^)	+2.3% (.72)	+9% (<.001)

^a^MVPA: morderate-vigorous physical activity.

^b^BAI: Beck Anxiety Inventory.

^c^STAI: State-Trait Anxiety Inventory.

^d^CESD: Center for Epidemiologic Studies Depression.

**Table 4:** Summary of descriptive statistics related to perceptions of participants’ own activity and their friend’s activity based on survey responses. *Change* refers to the percent difference between the average score for the first and last survey administered.

Category		Positive MVPA^a^ Trend	Negative MVPA Trend
		Change (*P* value)	Change (*P* value)
**Perception (higher score indicates a higher level of physical activity)**			
	Level of own activity	+2% (.17)	−8% (.01)
	Level of friend’s activity	+4% (.01)	−7% (.03)

^a^MVPA: moderate-vigorous physical activity.

**Table 5:** Summary of descriptive statistics related to perceptions of participants’ own physical activity and their friend’s physical activity based on survey responses stratified by presence of same-type alters in individuals’ ego networks. *Change* refers to the percent difference between the average score for the first and last survey.

Category		Positive MVPA^a^ trend		Negative MVPA trend	
		No PT^b^ alter (n=15), change (*P* value)	Has PT alter (n=26), change (*P* value)	No NT^c^ alter (n=17), change (*P* value)	Has NT alter (n=25), change (*P* value)
**Perception (higher score indicates a higher level of activity)**					
	Level of own activity	+4% (.03)	−1% (.98)	−10% (.05)	−2% (.31)
	Level of friend’s activity	−4% (.004)	+6.5% (.21)	−3% (.22)	−8% (.02)

^a^MVPA: moderate-vigorous physical activity.

^b^PT: positive trend.

^c^NT: negative trend.

## Discussion

### Principal Findings

In this paper, we presented PATX, a framework for capturing trends in MVPA habits using HR data from wearable fitness trackers. With a dataset featuring HR data across 2 academic years from a cohort of college students, PATX labeled 41 students as significantly increasing their MVPA overtime and 44 significantly decreasing their MVPA overtime. To demonstrate the value of these trends, 2 case studies were performed: the first, showing these trends could be used to support a body of previous work investigating the association between MVPA and mental health, and the second, establishing PATX’s value in supporting novel MVPA behavior change analyses such as the association among perceptions of individuals’ PA relative to their social networks.

In constructing our framework, we aimed to alleviate data quality issues related to fitness trackers including missing data and biases in measurement. By extracting trends in MVPA from the processed data, PATX offers a way to label individuals with increasing or decreasing trends. These trends provide a flexible manner to further examine changes in behavior, allowing for traditional statistical analyses to be performed on exposure variables among participants or groups.

Our first case study demonstrating this flexibility sought to support earlier work investigating the relationship between MVPA and mental health. Earlier work suggests engaging in MVPA may improve mental health, whereas abstaining may lead to complications [44-46]. Our observations conformed with these previous suggestions as participants with positive MVPA trends reported improved self-image, self-esteem and health, whereas participants with NTs increased in risk of anxiety and depression.

The second case study sought to examine how individuals perceive their own MVPA relative to their friends’ MVPA. For both MVPA trends, we observed that participants were *less* likely to report changes in their own MVPA when their close friends exhibited similar changes in MVPA. However, participants without friends exhibiting similar changes were *more* likely to report changes in their own MVPA. Although previous work investigating perceptions has shown individuals accurately perceiving their level of fitness, our findings suggest noticing changes in fitness may be more difficult when such changes are mirrored by close ties in one’s social network [47]. Therefore, to improve the efficacy of MVPA behavior change interventions, designs may need to be extended to incorporate one’s social circle.

By applying PATX to each of these case studies, we were able to utilize the objective and continuous measures of MVPA provided by fitness trackers across the entire period participants were under observation, providing a stronger representation of participants’ MVPA habits compared with measuring only intermittent weeks.

### Comparison With Previous Work

Although previous works have investigated long-term changes in MVPA, many have only measured their participants intermittently, typically administering fitness trackers to participants for 1 week at each assessment [15-20]. Although a more feasible method of data collection, such designs are prone to several biases.

First, intermittent weeklong measurements across multiple years depend heavily on which weeks are measured as seasonality must be accounted for with winter months likely to yield less MVPA than summer [48]. Studies with shorter intervals among assessments are also prone to this bias as follow-ups at the third, sixth, and ninth months will capture variations between these seasons. To account for this, when appropriate, we utilize seasonal trend decomposition using Loess to account for any seasonality [37].

Furthermore, weeks *when* activity data are collected are subject to social desirability bias as participants may exhibit short-term changes in behavior when they are aware their behaviors are being monitored [49]. The novelty of simply having these devices for a week may also bias behaviors as fitness trackers themselves have been shown to spur short-term activity changes [50]. Although longer-term continuous tracking studies may be subject to the same bias, research is still inconclusive as to whether these devices promote long-term PA changes [51].

Even though other methods have been proposed to gather insights from PA data, many extend behavior change to a broader scope [21]. Although this is important for investigating how PA changed, it answers a different question than that addressed in this paper. Our method is more focused in that we specifically aim to examine whether a significant increase or decrease in an individual’s MVPA took place over an extended period.

### Limitations

We note our sample size as a limiting factor of this study and address the potential selection bias introduced regarding participants included in this analysis as opposed to the excluded participants (Table 1). We compared the 123 participants included in the study and the 508 who were withheld to ensure our demographics were still reflective of the overall university demographic distributions. We found no significant differences in demographic distributions among age, gender, or race between our sample and the participants excluded.

Not all questions asked of participants were present in each survey, some questions were only asked in certain waves to prevent the surveys from extending beyond a reasonable duration for completion, which otherwise may cause participants to provide less accurate answers. Furthermore, although many within-study ties exist among participants in the NetHealth study, we were unable to capture any MVPA changes among an ego’s ties to participants outside the study, prohibiting a complete representation of MVPA changes throughout an ego network. As such, future studies may benefit from asking participants about those in their social network who cannot be directly observed to gather a more complete representation.

Finally, we note that given the nature of the NetHealth study, our sample has minor variation in age, truncated variation in socioeconomic background, and the fact that all participants were observed in the same environment. As a result, additional studies are necessary across different age groups and backgrounds to validate these findings.

### Conclusions

The transition from surveys to fitness trackers for measuring MVPA in behavior change studies delivers more objective assessments but introduces a new set of challenges. Data preprocessing steps and alternative means of analysis must be considered when using these devices. In this paper, we present a framework for navigating these data issues and extracting meaningful trends in MVPA from fitness trackers. With 2 case studies, we demonstrated the efficacy and flexibility of the outputs provided by our framework and how they can be used to support future MVPA behavior change studies.

## Abbreviations

ANOVA: analysis of variance
BAI: Beck Anxiety Inventory
CESD: Center for Epidemiologic Studies Depression
HR: heart rate
HRR: heart rate reserve
MVPA: moderate-vigorous physical activity
NT: negative trend
PA: physical activity
PATX: Physical Activity Trend eXtraction
PT: positive trend
RHR: resting heart rate
RMSE: root-mean-square error
SMS: short message service
STAI: State-Trait Anxiety Inventory
